# Microbiota and Human Reproduction: The Case of Female Infertility

**DOI:** 10.3390/ht9020012

**Published:** 2020-05-03

**Authors:** Rossella Tomaiuolo, Iolanda Veneruso, Federica Cariati, Valeria D’Argenio

**Affiliations:** 1 KronosDNA srl, Spinoff of Federico II University, 80133 Napoli, Italy; rossella.tomaiuolo@unina.it (R.T.); cariati@ceinge.unina.it (F.C.); 2 Department of Molecular Medicine and Medical Biotechnologies, Federico II University, Via Sergio Pansini 5, 80131 Napoli, Italy; io.veneruso@studenti.unina.it; 3 CEINGE-Biotecnologie Avanzate scarl, Via Gaetano Salvatore 486, 80145 Napoli, Italy; 4 Department of Human Sciences and Quality of Life Promotion, San Raffaele Open University, via di val Cannuta 247, 00166 Roma, Italy

**Keywords:** microbiota, human microbiome, human reproduction, female reproductive system, female infertility

## Abstract

During the last decade, the availability of next-generation sequencing-based approaches has revealed the presence of microbial communities in almost all the human body, including the reproductive tract. As for other body sites, this resident microbiota has been involved in the maintenance of a healthy status. As a consequence, alterations due to internal or external factors may lead to microbial dysbiosis and to the development of pathologies. Female reproductive microbiota has also been suggested to affect infertility, and it may play a key role in the success of assisted reproductive technologies, such as embryo implantation and pregnancy care. While the vaginal microbiota is well described, the uterine microbiota is underexplored. This could be due to technical issues, as the uterus is a low biomass environment. Here, we review the state of the art regarding the role of the female reproductive system microbiota in women’s health and human reproduction, highlighting its contribution to infertility.

## 1. Introduction

The availability of highly sensitive technologies, i.e., next-generation sequencing, for the in-depth study of microbial communities’ composition and richness, has prompted the interest into metagenomics. As a consequence, novel insights regarding the role of the microbiota in human physiology and pathology are gathering [1,2,3].

This also applies to the female reproductive system [4]. Indeed, for a long-time, culture-based approaches have been used to address the presence of microbes in this body niche and verify their possible implications in human reproduction. More recently, the Human Microbiome Project has assessed that the vaginal microbiota accounts for about 9% of the whole human microbiota [5,6,7]. In particular, *Lactobacilli* have been reported as the most represented bacteria in this body site, other represented genera being *Prevotella*, *Bifidobacterium*, *Gardnerella*, *Atopobium*, *Megasphaera*, *Sneathia*, and *Anaerococcus* [5,6,7]. These bacteria have been implicated in different phases of reproduction from gamete formation, fertilization, pregnancy establishment and maintenance, and in the microbial colonization of the fetus and/or of the newborn [3,4,8]. As a consequence, many efforts have been made to try to establish the composition and role of the “healthy” female reproductive system microbiome, and the effects of a dysbiosis on human reproduction and fertility [4,8].

This review aims to summarize the current knowledge regarding the female reproductive system microbiota composition and its role in women’s health. In addition, evidence suggesting a link between a dysbiosis of the female reproductive system microbiome and infertility will also be reviewed.

## 2. Materials and Methods

PubMed was searched for indexed articles in English published within 2010 and 2020. The following keywords were used: “female infertility and microbiota”, “female infertility and metagenomics”, “female reproductive system microbiome”, “uterine microbiome”, and “vaginal microbiome”. A manual search for the oldest references within the resulting articles was performed.

## 3. Female Reproductive System Microbiome

**Figure 1:**
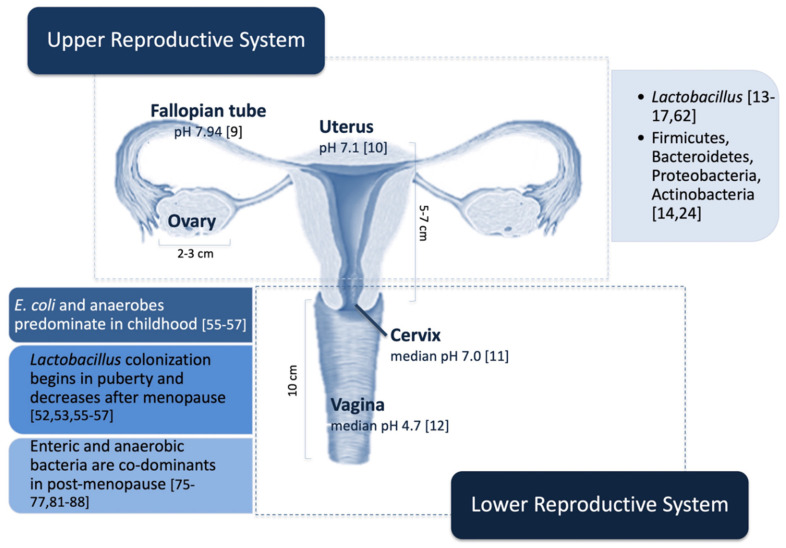
The female reproductive system microbiome in physiological conditions is not sterile but hosts a specific microbiota. In particular, the female upper reproductive system, which also includes the uterus and Fallopian tubes, hosts 10,000 times less bacteria than the vagina. Firmicutes, Bacteroidetes, Proteobacteria, and Actinobacteria have been reported as the most abundant phyla in the endometrium, the *Lactobacilli* being the most represented genus. The vaginal microbiota, under physiological conditions, undergoes fluctuations attributable to age, lifestyle, and environmental factors. The figure particularly emphasizes the modifications related to sex hormone levels. The pH values refer to average/median values found in physiological conditions, as pH also undergoes fluctuations due to several factors, including age, BMI, lifestyle, hormone replacement therapy, and microbiome alterations. References are reported in parentheses.

In the last years, increasing evidence has shown the presence of microorganisms not only in the vagina but also in the upper female reproductive system (including the ovaries, the Fallopian tubes, and the uterus), for a long time considered as a sterile niche [4]. Figure 1 summarizes the different bacteria reported so far in the different sections of the female reproductive system, as well as some organs’ details (size and pH) [9,10,11,12].

Interestingly, the *Lactobacillus* has been identified as the most abundant genus throughout all the female reproductive system [13,14,15,16,17]. These resident microbes contribute to health status maintenance, and their alterations have been associated with several gynecological diseases [18]. Indeed, *Lactobacilli* have been reported to exert protective effects respect to pathogens’ invasion, and a dysbiosis has been related to several diseases, including chronic endometritis, endometriosis, pelvic inflammatory disease, and gynecological cancers [15,18].

In this context, several studies have reported a positive correlation between the presence of specific bacteria in the uterus and the onset of pelvic inflammatory disease, an inflammation of the upper genital tract that can lead to infertility [19,20]. In addition, an increase of *Actinomyces*, *Corynebacterium*, *Enterococcus*, *E. coli*, *Fusobacterium*, *Gardnerella*, *Prevotella*, *Propionibacterium*, *Staphylococcus,* and *Streptococcus* has been associated with endometriosis [21,22,23]. Interestingly, Chen et al. found that the reproductive tract microbiome of women affected by endometriosis was different from that of women with infertility due to other causes [24]. If confirmed in other studies, this suggests that microbiota-specific alterations may lead to a better stratification of infertile women, and also to the development of different therapeutic strategies.

It is important to underline that an increasing number of studies are suggesting that female reproductive system microbes are worth exploring not just in the light of female general and reproductive health, but also of the health of the partner(s) and offspring.

First of all, Dominguez-Bello et al., reviewing the role of the microbiome in human development, highlighted that the host–microbiome superorganism seems to have coevolved to adapt to the environment and survive [25]. In this evolutionary context, the human microbiota has been suggested to be transferred throughout generations, via matrilineal, vertical crossover of bacteria, leading to the transmission of a phylogenetic mark [25]. As a consequence, the female reproductive system microbiota not only is important for women’s health but is able to influence the next generation from gestation [3].

Next, even if the uterus is an immune-protected organ, this does not mean constant sterility. In particular, it seems that cervical bacteria may enter with the sperm during fertilization and interact with the egg cells in the first step of embryonic development [25]. This bacterial transmission to the fetus seems to be critical for fertility, as assessed by the observation that gestational infections and inflammations reduce fertility and increase the risk of preterm birth [26]. The maternal microbiota may affect the fetus through several mechanisms, including direct effects (such as immune responses or bacterial metabolites able to cross the placenta) [27,28], and indirect factors able to act as epigenetic modifiers of the fetus (including diet, stress and neuroendocrine factors) [29,30,31].

Finally, it has been assessed that both gut and vaginal maternal microbiomes vary during pregnancy; however, the significance of these modifications for the mother and/or the newborn is still unclear [32,33]. Koren et al., analyzing the fecal microbiota of 91 pregnant women and their infants, showed that fecal microbiota changes from first to third trimester and, despite a wide interindividual variability, they found an overall increase in Proteobacteria and Actinobacteria, and a reduced richness; interestingly, the third-trimester microbiota was associated with greater inflammation and energy content, able to induce pregnancy-like metabolism in germ-free mice, and impacted pregnant women’s metabolisms similar to metabolic syndromes [32]. Blaser and Dominguez-Bello speculated that these pre-partum modifications of the maternal gut microbiota, through the increase of butyrate-producing bacteria, may allow immune tolerance in the mother [34]. A recent study by Farr Zuend et al. highlighted cervicovaginal proteome and microbiome modifications in pregnant, with respect to non-pregnant, women and speculated that these alterations may be related to the increased risk of HIV infection during pregnancy [35]. Further studies are required to clarify how these bacterial modifications can impact women’s health and may promote or disadvantage women’s fertility.

These data not only underline the role of the resident microbiota for female health’s acquisition and maintenance but also suggest that the microbiota may be a novel, important target for the development of specific diagnostic tests or therapies.

### 3.1. Upper Reproductive System

Only recently, metagenomic studies have shown that the female upper reproductive system, which includes the ovaries, the Fallopian tubes, and the uterus (Figure 1), is not sterile but hosts a specific microbiota [4,8,13,14,15,16,17,18].

About 30 years ago, bacteria were cultured for the first time in endometrial samples [36]. Later, Mitchell et al. demonstrated the presence in the uterus of specific bacteria, different from those present in the vagina, even if the richness was significantly lower in the uterus [13]. According to the latter, Chen et al., by using both quantitative PCR and next-generation sequencing, revealed that the upper reproductive system hosts 10,000 times less bacteria than the vagina [24]. This lower bacterial load may be due to (i) the cervical barrier that may hamper the ascension of microbes from the vagina, (ii) specific immune reactions, and (iii) environmental factors prompting the growth of specific taxa [4]. Despite this low biomass, the uterine microbiota seems to be active: Firmicutes, Bacteroidetes, Proteobacteria, and Actinobacteria being the most abundant phyla [14,24]. In addition, *Lactobacilli* have been found to be the most represented genus in the endometrium [13,14,37].

This microbiota has been proposed to be able to modulate the functions of both endometrial cells and the local immune system and to prevent uterine infections by both competing with pathogenic microorganisms and producing protective molecules [38,39].

However, due to the difficulties in obtaining upper reproductive system samples from healthy women, few data are available to date. Moreno et al., comparing the endometrial and vaginal microbiome of fertile and healthy women, confirmed the prevalence of the *Lactobacillus* and found similar microbiome profiles between the two analyzed body sites in 80% of the cases [14]. Similar results were achieved by Kyono et al., even if in a small population [15].

Taken together, these data support the hypothesis of a continuum along the reproductive system with the ascension of microbes from the vagina; in addition, a microbial spreading from other organs through the blood has also been suggested [40,41].

However, it is noteworthy that there is still an ongoing debate on whether the upper organs of the female reproductive tract harbor bacteria. Indeed, several studies have used next-generation sequencing methods to assess the presence of bacteria in the placenta and correlate them to different health conditions [41,42,43]. On the other hand, other studies, taking into account contamination biases due to difficulties in detecting microbes in low-biomass samples, did not detect bacteria in the placenta [44,45,46,47]. De Goffau et al. analyzed 537 placental biopsies (318 with adverse outcome and 219 controls) to determine whether pre-eclampsia, delivery of a small-for-gestational-age infant, and spontaneous preterm birth were associated with the presence of specific bacteria, supporting the hypothesis of a placental microbiome [48]. They found that (i) placental samples had very low biomass and most of the bacteria were contaminants; (ii) some bacteria colonize the placenta only during labor and delivery, or are the result of ascending uterine infections; and (iii) the *Streptococcus agalactiae* was identified as a taxon of placental origin, even if it seems to be not related with the different pregnancy outcomes included in this work [48]. In addition to identifying an alternative route of *Streptococcus agalactiae* transmission to the infants, this study sheds light on a possible route of fetal colonization already in utero, through the placenta. Perez-Munoz et al. highlighted how evidence supporting the “in utero colonization hypothesis” is still extremely weak, as the used molecular approaches have a detection limit insufficient to analyze “low-biomass” microbial populations, contamination controls are often not taken into account, and bacterial viability is not proven [49]. Indeed, low-biomass samples are extremely difficult to analyze and well-designed, case-control studies on large cohorts of samples, taking into account laboratory contamination sources and other technical limits [50], are required to address the above-mentioned issues. This will allow to establish the functions of the female upper reproductive system microbiome and assess its role in the context of its host, partner, and offspring.

### 3.2. Lower Reproductive System

The vaginal microbiota is the most studied within the female reproductive system (Figure 1). Indeed, vaginal microbiota has been related to vaginal health preservation and host defense against diseases [51]. In healthy, reproductive-age women, the vaginal microbiome accounts for about 1 billion bacteria/gram of vaginal fluid and is featured by low biodiversity, *Lactobacillus* species accounting for up to 95% of total bacteria [52,53]. Despite this well-defined composition, there is a certain consensus that vaginal microbiota undergoes important composition fluctuations during women’s life, sex hormones playing a key role in this scenario [51,54]. Interestingly, the *Lactobacillus* predominance seems to be age-dependent and strictly related to the reproductive age: in childhood *Escherichia coli* and anaerobes predominate, with puberty *Lactobacillus* colonization begins, and finally its abundance decreases after menopause [55,56,57]. In particular, in post-menopausal women, the estrogen level reduction causes an increase of vaginal pH that allows the colonization of enteric bacteria and anaerobes that are co-dominant, vary among women, and resemble those found in women with bacterial vaginosis [51,58]. Indeed, bacterial vaginosis is the most common form of vaginal dysbiosis and is a condition featured by the shift of vaginal microbiota from *Lactobacilli* to facultative anaerobe. It is important to underline that the presence of *Lactobacilli* in the lower female reproductive system has also been related to racial, genetic, geographic, and social factors [59]. Finally, the vaginal microbiota is influenced by several environmental factors, such as hygiene habits, sexual exposure, change of sexual partners, and use and type contraceptives, that may be responsible for microbial fluctuations over time [60,61,62].

Despite all these variables, *Lactobacillus* spp. are predominant in this body niche in almost all women, and play an important role in the maintenance of vaginal homeostasis, as underlined by the findings that the depletion of these bacteria has been associated to several diseases [63]. In particular, *Lactobacillus crispatus*, *Lactobacillus gasseri*, *Lactobacillus iners,* and *Lactobacillus jensenii* are the most represented in the vagina of most healthy women and is yet unclear why other species, such as *Lactobacillus helveticus* or *Lactobacillus acidophilus*, are not present [63,64]. Ravel et al. analyzed 396 reproductive-age women and, based on the abundance of the identified *Lactobacillus* spp., distinguished 5 bacterial profiles [64]. Interestingly, while 4 bacterial profiles (accounting totally for 73% of the analyzed women) were dominated by a different *Lactobacillus* spp. (*L. crispatus*, *L. gasseri*, *L.s iners,* and *L. jensenii*), only one of these profiles was non-*Lactobacillus*-dominated and was featured by the presence of strictly anaerobic bacteria, such as *Atopobium*, *Dialister*, *Gardnerella*, *Megasphaera*, *Prevotella*, and *Peptoniphilus* [64]. The role of these bacteria in maintaining vaginal health is still under debate [59,60]. Indeed, even if these bacteria have been identified in asymptomatic women, they have also been related to a high Nugent score, a common mark of bacterial vaginosis, and has also been related to an increased risk of sexually transmitted diseases and adverse pregnancy outcomes [65].

Dominant *Lactobacilli* maintain vaginal homeostasis by several direct and indirect antipathogenic mechanisms, such as the formation of microcolonies that, by their adhesion to the epithelial cells, are able to create a physical barrier against pathogens’ adhesion, and/or the activation of immune reactions against pathogens [63,66,67,68]. The production of lactic acid, by lowering the pH, is one of these mechanisms and is able to inactivate or kill several pathogens [66,67]. In addition, hydrogen peroxide has also been shown to damage vaginal pathogens and may increase pathogen sensitivity to antibiotics [68].

These molecular mechanisms reinforce the role of *Lactobacillus* spp. in vaginal health maintenance. As mentioned above, vaginal bacterial dysbiosis is featured by the reduction of *Lactobacilli* and the increase of anaerobic microorganisms. These modifications are able to produce amino compounds and increase vaginal pH, thus creating an environment more prone to pathogenic infections and more susceptible to unhealthy conditions, including reproductive outcomes [69]. To date, a number of factors, including acquired and modifiable factors (such as diet, tobacco smoke, stress, hygiene practices, sexual habits, use of oral contraceptives, and assumption of probiotics and/or antibiotics), have been associated to vaginal dysbiosis. This allows us to hypothesize that the more our knowledge in this field increases, the more it will be possible to design specific interventions (such as probiotics assumption) that, targeting the vaginal microbiota, will be able to prompt women’s health during their lifetimes and reduce the risk of specific diseases onset. As mentioned before, a better understanding of the female reproductive system microbiome and their physiological functions will also help to clarify its contribution to partners’ and offspring’s health.

## 4. Female Reproductive System Microbiome and Infertility

Infertility incidence has so increased in the last years that it is currently recognized as a worldwide health issue [70]. One in seven couples is affected by infertility, defined as the inability to conceive after 1 year of regular unprotected intercourse. The first steps of the infertility journey are to investigate the presence of ovulation, uterine cavity anatomy, and patent fallopian tubes in women, and semen parameters in men. The female factors, responsible for 35%-40% of couples’ infertility, can depend on several causes, including hormonal changes, tubal changes, uterine pathologies, maternal age, and systemic or genetic diseases. However, about 35% of couples, despite the above-mentioned clinical and instrumental assessment, still remain with no diagnosis [70]. In vitro fertilization (IVF) techniques offer several opportunities based on the causes and severity of infertility; however, the molecular mechanisms underlying infertility are often unknown, leading to long diagnostic paths and ineffective therapies [70]. Indeed, despite the difficult-to-determine success rate of IVF procedures (because of the indices and patient populations used and/or selection biases and misunderstood statistics), the percentage of implantation and pregnancy rates per embryo transfer still remains low [71,72].

Today, the challenges facing scientists are to recognize the hidden causes of infertility and to improve the efficiency and the efficacy of IVF techniques. Taking into account the previous considerations, the microbiome has been recently considered as a topic of interest for infertility.

**Figure 2:**
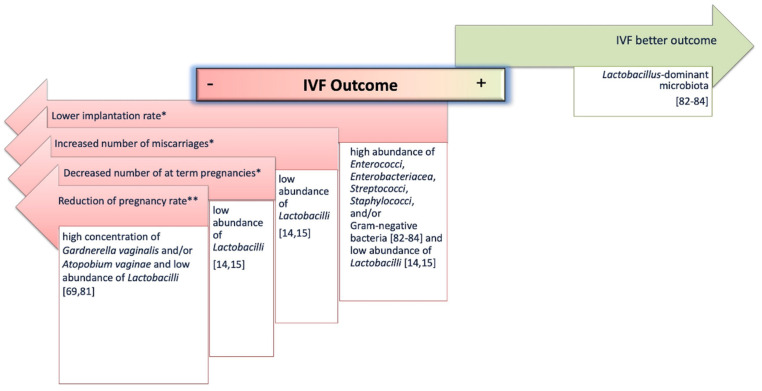
Female reproductive system microbiome alterations have been associated with different IVF outcomes. While *Lactobacilli* spp. have been reported to exert beneficial effects (green arrow), specific endometrial * or vaginal ** dysbiosis has been related to a worse success rate (pink arrows). References are reported in parentheses.

Accordingly, an increasing number of studies is highlighting a correlation between infertility and the microbiota [4,63,73,74,75,76,77]. Indeed, it has been described that infertile women host a different microbiota, both in the lower and/or in the upper reproductive system, with respect to fertile women [4,63,73,74,75,76,77]. Campisciano et al. analyzed the vaginal microbiome of idiopathic infertile women to highlight specific alterations with respect to that of healthy, bacterial-vaginosis-affected, and non-idiopathic infertile women [75]. They not only found a significant clusterization of the four groups but were also able to identify specific bacterial taxa able to distinguish the idiopathic infertile women from the others [75]. Wee et al. retrospectively analyzed vaginal, cervical, and endometrial samples from infertile versus fertile women [76]. This pilot study found that *Ureaplasma* and *Gardnerella* were more abundant, respectively, in the vagina and in the cervix of the infertile women [76]. Moreno and Simon recently reviewed the importance of endometrial microbiota for women’s health with a special focus on infertility [77]. In addition, it has been suggested that IVF procedures’ outcomes may be affected by the resident microbiota (Figure 2).

### 4.1. Female Reproductive System Microbiome and IVF Outcomes

Taking in to account systematic reviews and meta-analysis data, negative predictors of IVF seems to be female age, duration of infertility, and basal FSH levels [78]. Contrasting data exist about the anti-Müllerian hormone level [79,80]. On the contrary, a high number of oocytes and embryo quality are positively associated with good IVF outcome [78]. Nevertheless, there is no significant increase in pregnancy rates after IVF procedures [71,72]. Thus, there is an increasing interest in the identification of additional factors able to affect IVF success rates. In this context, the potential effects of the microbiota on IVF outcomes are currently under investigation, as detailed afterwards.

In particular, alterations of the vaginal microbiota have been correlated to a significant reduction of the pregnancy rate after IVF [69,81]. Haar et al., analyzing the vaginal microbiota of 130 IVF patients, found that only a small percentage of those with a vaginal dysbiosis, defined by a high concentration of *Gardnerella vaginalis* and/or *Atopobium vaginae,* had a clinical pregnancy, suggesting that vaginal dysbiosis may negatively impact IVF pregnancy rates [81].

Moreover, the association between endometrial microbiome alterations and IVF failure has also been investigated [14,15,37,82,83,84,85]. Indeed, the culture of the tips of the catheter used for embryo transfer has revealed that, while the presence of *Lactobacilli* is associated to a better reproductive outcome, the isolation of *Enterococci*, *Enterobacteriaceae*, *Streptococci*, *Staphylococci*, and/or Gram-negative bacteria is correlated with lower implantation rate, decreased number of at-term pregnancies and increased number of miscarriages [82,83,84]. Accordingly, Moreno et al. found that about 46% of IVF women with a receptive endometrium had a non-Lactobacillus-dominant microbiota and assessed that this microbial profile was associated with a poor reproductive outcome in terms of lower implantation rates, pregnancy rates, ongoing pregnancies, and live birth rates [14]. In addition, Kyono et al. found a different and progressively reduced abundance of *Lactobacilli* between healthy volunteers, non-IVF patients, and IVF patients (85.7%, 73.9%, and 8% of relative abundance, respectively), showing that a high number of IVF patients harbor a dysbiotic microbiome at the endometrial level and that a high content of *Lactobacilli* may improve the implantation rate [15]. However, Franasiak et al., by using 16S rRNA sequencing, identified *Lactobacillus* and *Flavobacterium* as the most abundant genera on the tips of the catheter used for embryo transfer but were not able to find a statistically significant association among these taxa and patients’ IVF outcomes [37]. Recently, Hashimoto et al. analyzed the pregnancy outcomes of 99 IVF patients presenting eubiotic or dysbiotic endometrium at the time of embryo transfer to verify what bacterial profiles are related to the better embryo implantation rate; however, they found no significant differences [85].

All the above highlight the need for further studies to try concluding remarks on the relationship between endometrial dysbiosis and IVF outcomes. If a significant association is confirmed, this will open the way to therapeutic options based on microbiome modifications. Indeed, it is suitable to hypothesize that the modulation of the uterine microbiome, i.e., the increase of *Lactobacilli* and the decrease of anaerobe microbes (such as *Atopobium vaginae, Gardnerella vaginalis, Propionibacterium acnes, and Streptococcus agalactiae*) [86], may improve IVF outcomes. In this context, the use of antibiotics is still controversial since no beneficial effects of antibiotic administration before embryo transfer have been reported on pregnancy outcome [87]. However, Vitagliano et al., evaluating the efficacy of antibiotic administration in the treatment of chronic endometritis, found that the treatment not only was effective in eliminating the cause of the infection but had a positive impact on the implantation and pregnancy rate and improved IVF success [88]. Similarly, Cicinelli et al. found that chronic endometritis was prevalent among women with unexplained infertility, and that antibiotic treatment improves spontaneous pregnancy rates and live birth rates in these patients [89].

The use of probiotics may also be useful in these patients; indeed, several oral and vaginal probiotics containing *Lactobacillus* spp. are available on the market. However, the efficacy of these treatments, alone or coupled with antibiotics, is still under investigation [90,91,92,93]. A recent study by Chenoll and colleagues investigated the effects of *Lactobacillus rhamnosus* BPL005 on the improvement of the female reproductive tract and were able to assess the protective role of this strain on endometrial infections in an in vitro model of bacterial colonization of primary endometrial epithelial, suggesting its potential use in gynecological conditions [86].

### 4.2. Partner’s Semen Microbiota Contribution and Infertility

While several data are available regarding female infertility, male infertility factors are relatively understudied, and the available data are often contrasting. The male factors considered as potential predictors of IVF outcome include sperm quality parameters and DNA fragmentation [94,95,96]. In addition, a meta-analysis, including a total of 2906 subjects evaluated in 34 prospective studies, showed that non-conventional assays, such as sperm-zona pellucida-binding and the induced-acrosome reaction, have high predictive power for fertilization outcome [97].

In addition to all the above, it is important to underline that increasing evidence has addressed that to conceive a baby, the reproductive systems of both partners need to work in a proper coordinate manner [70]. This also applies to the microbiome, the semen microbiome being able, through microbial transfer, to affect both couples’ and newborns’ health [98]. In this context, Mändar et al. analyzed semen and vaginal microbiomes of 23 couples to assess the effects of sexual intercourse on vaginal microbiome [99]. Interestingly, they found that the prevalence of *Gardnerella vaginalis* in the vaginal microbiome was higher in women whose partners had leukocytospermia; in addition, they also highlighted a reduction of *Lactobacillus crispatus* and a high concordance between semen and vaginal microbiome, after sexual intercourse [99]. Subsequently, Amato et al. analyzed the vaginal and seminal microbiome of 23 couples with idiopathic infertility with the aim to correlate microbial features with the pregnancy rate after intrauterine insemination [100]. They found that the vaginal microbiome of idiopathic infertile women differed from controls, whereas the semen not; in addition, they found, among the vaginal microbiome of idiopathic infertile women different patterns of *Lactobacillus* species, *Lactobacillus crispatus* being associated to the higher rate of intrauterine insemination success [96]. More recently, Štšepetova et al. analyzed the microbiome of native semen samples used for IVF, processed semen samples, and IVF culture media, with the aim to associate them with IVF embryo quality and pregnancy rates [101]. The study highlighted several bacterial changes in the IVF samples; in particular, the presence of *Staphylococcus* spp. and *Alphaproteobacteria* may influence sperm and embryo quality, suggesting that methods able to reduce the effects of these bacteria on IVF embryo development may avoid IVF failure [101]. All these data strongly support the hypothesis that semen acts as a medium for the transmission of microorganisms that may potentially become residents in the uterus [102]. In addition, it has also been proposed that males can transmit information to their partners and progeny through the microbiome [103]. Further studies are required to assess the role of this “seminovaginal” microbiome in couples’ health, its alterations in infertility, and its ramifications to the offspring.

This strict association may also be used to design targeted therapeutic approaches. For example, Plummer et al. analyzed 22 couples to assess the effect of topical and oral antimicrobial therapy in both women with bacterial vaginosis and their male partners on vaginal and penile microbiomes [104]. Their data showed a reduction of bacterial diversity and the reduced abundance of taxa related to bacterial vaginosis in the vaginal microbiome; the composition of the cutaneous penile microbiome was also modified even if these changes were not stable over time [104]. This study suggests that a combined oral and topical therapy of male partners of women affected by bacterial vaginosis may be effective.

Taken together, these studies highlight the importance of the female reproductive system microbiome for human reproduction, suggesting that microbial dysbiosis not only may be associated with infertility but may also play a role in IVF outcomes. Thus, its evaluation should be taken into account, together with the male semen microbiome, when assessing infertile couples.

## 5. Conclusions

**Figure 3:**
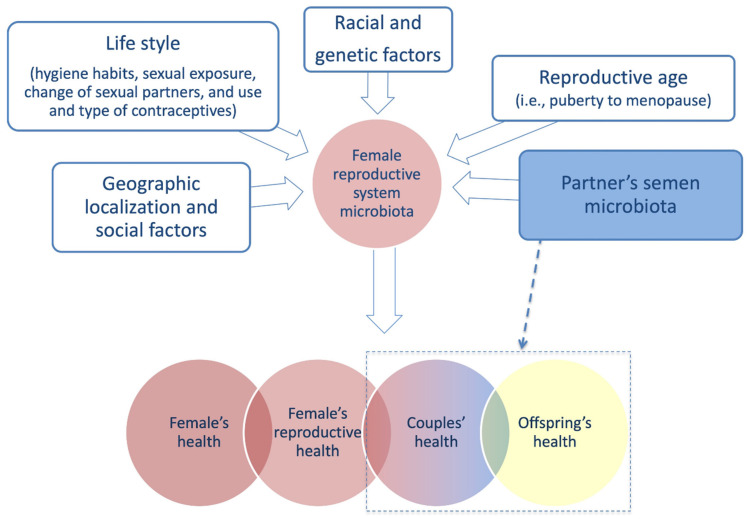
Individual variability factors affecting female reproductive system microbiota composition and processes in which it has been suggested to play a role [54,55,56,57,58,59,60,61,62,63,73,74,75,76,77]. The evaluation of the partner’s semen microbiota composition is important as it has implications not only on the composition of the female one but also on the reproductive health of the couple and offspring [105].

An increasing number of studies are underlying the importance of the female microbiota in human reproduction and fertility. As for other metagenomic fields, some technical issues have to be carefully taken into consideration in order to minimize potential biases and obtain concluding remarks [49,51]. The uterine microbiota, in particular, due to its low biomass and the difficult sampling (especially in healthy controls), is challenging to be studied. In addition, several internal and external factors can affect the female reproductive system microbiome and have to be taken under consideration to try to minimize intra- and inter-group variability (Figure 3).

Finally, to correctly evaluate the reproductive status of a couple, the microbiota evaluation of the male partner is mandatory [70]. It has been recently stated that the reproductive systems of both partners work in a coordinate manner: this sentence also applies to the so-called “seminovaginal” microbiome, meaning a functional unit able to influence not only the couples’ health and their reproductive outcomes but also their offspring’s health [99,105]. Further studies are required to definitively assess the role of this seminovaginal microbiome in infertility and also in IVF outcomes in order to improve the pregnancy rate. These data may support the utility to test the microbiota in couples’ infertility assessment in order to plan the most proper and personalized treatment. Indeed, the microbiome offers a unique opportunity to develop specific treatments aimed at its modification. Several issues still remain to be clarified and, once our knowledge in this field increases, this will open the way to novel opportunities for infertility management and treatment.
